# Vernal Keratoconjunctivitis: Immunopathological Insights and Therapeutic Applications of Immunomodulators

**DOI:** 10.3390/life14030361

**Published:** 2024-03-09

**Authors:** Navpreet K. Hehar, DeGaulle I. Chigbu

**Affiliations:** Pennsylvania College of Optometry, Salus University, Elkins Park, PA 19027, USA; dchigbu@salus.edu

**Keywords:** Th2 cells, IL-4, IL-5, IL-13, vernal keratoconjunctivitis, immunomodulators, cyclosporine A (CsA), tacrolimus

## Abstract

Vernal keratoconjunctivitis (VKC) is a complex and multifactorial disease process that employs Th2 cell-mediated immunologic processes, which involves the overexpression of interleukin 4 (IL-4), IL-5, IL-9, IL-13, and IL-31, and the activation of mast cells that release IL-5 and CCL-11, recruiting eosinophils to the site of inflammation. The disease primarily affects young males and is more common in regions with warm climates. VKC is characterized by persistent and recurrent conjunctival inflammation that can adversely affect the patient’s quality of life, and, when inadequately treated, may lead to a host of ocular complications, such as corneal shield ulcers and scarring. The major distinct forms of VKC include limbal or palpebral, which may occur in combination. The clinicopathological features of VKC include the presence of pseudogerontoxon, limbal gelatinous hyperplasia, and perilimbal hyperpigmentation. Topical immunomodulators are effective anti-steroidal options for controlling severe and chronic cases of VKC. This review will provide a brief overview of topical immunomodulators, including cyclosporin and tacrolimus, and will highlight the clinical manifestations, pathological mechanisms, and fibroproliferative changes in the conjunctiva that can result from recurrent disease.

## 1. Introduction

Vernal keratoconjunctivitis (VKC) is a chronic, and potentially severe, allergic inflammatory disease that affects the ocular surface [1,2,3]. Although the name “vernal” implies a seasonal pattern, with repeated bouts of inflammation occurring during warm seasons, VKC becomes perennial in approximately 25% of patients who experience symptoms throughout the year [1]. VKC is classified into three distinct categories based on the ocular area that is predominantly affected: namely, either limbal, palpebral, or a mixed form of VKC [1,4,5]. The palpebral form of VKC is characterized by the presence of giant papillae on the upper tarsal lid, while the limbal form is characterized by the presence of gelatinous limbal infiltrates, and Trantas dots [6]. Transient yellow-white deposits with an opaque appearance may be present in limbal VKC and are termed Horner–Trantas dots [1,7]. Histological examination reveals epithelial cell debris with dense infiltration of eosinophils [1,8,9]. In the mixed form, both the upper tarsal lid and limbus are involved. The etiology of this condition is considered to be a combination of type I and type IV hypersensitivity reactions that are characterized by an overexpression of mast cells, eosinophils, Th2 cells, cytokines, and chemokines, which play a significant inflammatory role [1,4,5]. This inflammatory cascade contributes to the characteristic symptoms and ocular surface changes seen in VKC, which include intense pruritis, redness, photophobia, ropy mucus discharge, and foreign-body sensations [1]. Pathognomonic signs of VKC include the presence of yellow-white limbal deposits, known as Horner–Trantas dots, cobblestone giant papillae of the upper tarsal lids, and shield ulcers [1,6]. Additional ocular signs observed in patients with VKC include perilimbal conjunctival pigmentation, punctate epithelial keratitis, and pseudogerontoxon [1,6].

Management of VKC involves a combination of topical therapy including mast cell stabilizers, antihistamines, NSAIDs, corticosteroids, or immunomodulators [4,5,9]. Immunomodulators have been used in clinical practice since 1959 [10]. The utilization of these agents is prevalent in ophthalmic care for addressing a spectrum of ocular conditions including dry eye disease [11,12], allergic conjunctivitis [13], non-infectious uveitis [14,15], and other recalcitrant ocular surface diseases [16]. Immunomodulator therapy offers new therapeutic options in the management of chronic inflammatory conditions as they are steroid-sparing agents. Topical immunomodulators are well tolerated, have a better safety profile, and play a crucial role in the management of moderate to severe allergic eye disease.

This paper will review the disease process of VKC, including the clinicopathological features of VKC, and discuss the anti-inflammatory and immunomodulatory agents that can be used to treat moderate, severe, or recurrent forms of the disease. While VKC is typically not sight-threatening, its impact on the patient’s quality of life can be substantial. Understanding its clinical features, underlying mechanisms, and appropriate treatment strategies is crucial in order to provide symptomatic relief and prevent serious ocular sequelae.

## 2. Epidemiology of VKC

VKC is characterized by persistent allergic inflammation that affects the conjunctiva and culminates in acute symptoms, including intense pruritis and ropy mucus discharge. Patients may also complain of ocular irritation, photophobia, foreign-body sensations, and excessive tearing. The epidemiology of VKC reveals certain patterns regarding its demographic distribution and its prevalence. VKC has an extreme geographical distribution but is more prevalent in regions with warm and dry climates, such as Central and South America, West Africa, the Indian and Mediterranean subcontinent, the Middle East, and parts of Asia [1,2,3]. This particular ocular disorder primarily affects pre-pubescent individuals, wherein the majority of cases have been reported to occur in the first decade of life [1,2]. While most cases of VKC resolve after puberty, some cases may persist into adulthood [17]. Interestingly, the great majority of patients that develop VKC are male, with the male-to-female ratio reported to be from 2:1 to 4:1 [1,2]. This male preponderance dissipates after puberty, where both genders are affected almost equally [1]. VKC is commonly a bilateral condition (98%) and can occur alone or be associated with atopic diseases such as asthma, allergic rhinitis, and eczema [1,3]. Sex-related hormonal influences have been postulated in the pathogenesis of VKC due to the observation of a predilection for males, with spontaneous recovery after puberty [18,19]. Estrogen and progesterone receptors are found to be overexpressed in the epithelium of the tarsal and bulbar conjunctiva in VKC patients and are reported to modulate the activity of eosinophils, specifically resulting in degranulation of eosinophils [18,20]. The role of the endocrine system may explain the recurrent nature of this disease in the adult population. Recurrent VKC in adulthood is different from adult VKC-like disease, which is described as a new onset after puberty with signs and symptoms similar to those in the pediatric form of the disease, but with a lower male-to-female ratio, lower incidence, and less corneal involvement [18,19]. VKC in adults is not common and is reported to account for 12% of VKC cases [21]. Adult patients with recurrent VKC tend to have more severe inflammatory reactions compared to childhood VKC patients, which has been confirmed by the increased level of acute inflammatory markers present in this adult form [22,23]. As such, these patients are at a higher risk of developing severe chronic complications of VKC, including scarring, corneal ectasia, and limbal stem cell deficiency; therefore, this dictates the increased need for topical corticosteroids and immunosuppressive therapy to adequately manage the inflammation [22,23].

## 3. Immunopathophysiology of VKC

The immunopathogenesis of VKC involves a complex interplay of the immune system that leads to the activation of an exaggerated immune response to specific allergens. Exposure of susceptible individuals to dust, pollen, and other wind-borne allergens is thought to contribute to conjunctival hyper-reactivity and lead to an inflammatory cascade [5]. VKC is classically considered a type IV hypersensitivity reaction, in which Th2 lymphocytes produce pro-inflammatory mediators including IL-3, IL-4, IL-5, IL-9, IL-13, and IL-31. These Th2 cell-derived cytokines are responsible for the clinicopathological expression in the conjunctivae and corneas of patients with vernal keratoconjunctivitis. Additionally, IL-9 is produced by Th9 cells and mast cells [24]. As such, Th2 and Th9 cells have a role to play in the immunopathophysiology of VKC.

There is an overexpression of IgE that primes the conjunctival mast cells as well as the activation and recruitment of eosinophils [1,5,9,25]. Histopathological analysis reveals conjunctival infiltration with immune cells, including eosinophils, lymphocytes, and basophils, which produce inflammatory mediators that promote tissue damage and post-inflammatory tissue remodeling [5,25]. Epithelial cells, mast cells, and fibroblasts are present in the conjunctiva, and these cells play a role in the immunopathophysiology of vernal keratoconjunctivitis. Epithelial cells express receptors for IL-9 [26,27], IL-33 [28], and histamine [29,30], and as such, epithelial cells can participate in mediating the immune and pathological changes that occur in the conjunctivae and corneas of individuals with vernal keratoconjunctivitis [4].

In a murine model of allergic experimental conjunctivitis, it was shown that IL-9 bound to IL-9R expressed on conjunctival and corneal epithelial cells induced a breach of the barrier function of the epithelium of the ocular surface [31]. This can manifest as punctate epithelial erosions due to the breakdown of the tight and adherent junctions that link the individual epithelial cells of the ocular surface. Furthermore, the breach of the epithelium can facilitate the access of allergens to antigen-presenting cells resident in the subepithelial layer of the conjunctiva to promote type 2 allergic immune responses in the conjunctiva. Additionally, allergens can induce the crosslinking of IgE-attached receptors on sensitized conjunctival mast cells, leading to activation and degranulation of the mast cells and resulting in a type 2-mediated immune response during allergic inflammation of the conjunctiva [29,32,33,34].

A dysfunctional epithelial barrier of the cornea can provide a portal for immune mediators in the tear film to gain access to the corneal stroma. Cytokines such as IL-4 and TNF-alpha can activate the corneal fibroblasts to produce matrix metalloproteinase (MMP) that breaks down the corneal stroma. Additionally, MMP released from degranulated eosinophils and activated ocular surface epithelial cells can degrade the collagen matrix of the corneal stroma, leading to the development of a sterile corneal ulcer [4,9]. Conjunctival epithelial cells can act as a mediator of type 2 immune responses during allergen-induced conjunctival inflammation since activated epithelial cells of the conjunctiva can produce cytokines such as IL-25, TSLP, and IL-33 [35]. IL-33 produced by epithelial cells and fibroblasts of the ocular surface cells promotes the chronic inflammatory process that is characteristic of vernal keratoconjunctivitis, because it can activate mast cells and eosinophils during type 2 immune responses in allergic diseases [36,37,38,39]. Furthermore, IL-33 has been demonstrated to induce barrier dysfunction of ocular surface epithelium [31]. TSLP is a cytokine derived from conjunctival epithelial cells. It activates dendritic cells to promote the generation of Th2 cells that mediate type 2 allergic immune responses [40], as well as activating mast cells to secrete IL-4, IL-5, TNF-alpha, and IL-13 [41]. As such, conjunctival epithelial cells, along with IL-25, TSLP, and IL-33, have a role to play in the immunopathophysiology of chronic allergic disorders of the ocular surface, such as vernal keratoconjunctivitis.

Connective tissue mast cells contain tryptase and chymase, and they are usually located in the subepithelial layer of the conjunctiva and skin [42]. Histamine, proteases, and heparin are preformed mediators released by mast cells [43]. Histamine interacts with its receptors to induce various clinical expressions observed in individuals with VKC. Mast cell-derived tryptase can activate C3 and C5 to generate C3a and C5a, respectively [44]. C3a and C5a generated by the mast cell-derived tryptase can also mediate the recruitment of eosinophils to the site of allergic inflammation [45]. Fukuoka et al. [46] demonstrated that the complement components C3 and C5 are produced by human connective mast cells located in the skin. TNF-alpha and IL-4, or TNF-alpha and IL-13, can induce fibroblasts to secrete C3 [47]. The results of these studies have shown that connective tissue mast cells and fibroblasts can secrete C3. Protease secreted by domestic dust allergens can generate C3a from C3, and the generated C3a can interact with C3aR on mast cells to induce the activation and degranulation of mast cells with the consequential release of histamine, lipid mediators, chemokines, and cytokines [48,49,50]. This demonstrates that C3a generated via immune and non-immune mechanisms during the activation of the complement system can play a role in the immunopathophysiology of vernal keratoconjunctivitis [51].

Leukotriene B4 (LTB4), leukotriene C4 (LTC4), platelet-activating factor (PAF), prostaglandin D2 (PGD2), and prostaglandin E2 (PGE2) are lipid mediators released by mast cells [43]. Lipid mediators such as leukotrienes (LTB4 and LTC4) and prostaglandins (PGD2 and PGE2) induce changes in the diameter (vasodilation) and permeability (increased vasopermeability) of conjunctival blood vessels during allergic inflammation of the conjunctiva [52,53,54]. Lipid mediators play a role in promoting the influx of immune cells such as eosinophils to the conjunctiva during allergic inflammation [54]. PAF [55,56], PGD2 [57], and LTB4 [58,59] mediate the recruitment of eosinophils to the conjunctiva. The pro-inflammatory mediators released by eosinophils cause inflammation, damage, and remodeling of the ocular surface in VKC [7,53,60]. 

Cytokines (IL-4, IL-5, IL-9, IL-13, IL-25, IL-31, and IL-33) and chemokines (CCL5, CCL11, CCL17) are released by degranulated mast cells [4,43]. Conjunctival fibroblasts express receptors for IL-4 and IL-13. The binding of IL-4 or IL-13 to their receptors expressed on fibroblasts in the conjunctiva results in the proliferation and excess production of extracellular matrix by these activated conjunctival fibroblasts [61]. The overgrowth of fibroblasts in the conjunctiva during allergic inflammation results in the development of papillae [4,61]. Because IL-4- or IL-13-activated conjunctival fibroblasts do not undergo apoptosis by virtue of their activation of the phosphatidylinositol-3-kinase (PI3K)/AKT signaling pathway, these cytokines contribute to the development of papillae in vernal keratoconjunctivitis [61]. Similar to IL-4 and IL-13 [62,63], IL-9 can promote the production of IgE-secreting plasma cells [64,65]. IL-9 induces the upregulation of the IL-5R alpha chain on eosinophils [66]. IL-5 induces the proliferation, maturation, differentiation, and chemotaxis of eosinophils to the site of allergen-induced inflammation in the conjunctiva [4,29,67]. IL-25 can exacerbate the inflammatory process because of its ability to recruit eosinophils to the site of allergic inflammation [68]. CCL5 and CCL11 recruit eosinophils [69] and recruit Th2 cells [70] to the conjunctiva during the allergic inflammatory process to mediate the immunopathology of VKC [4]. As such, lipid mediators, cytokines, and chemokines play a role in the immunopathophysiology of VKC.

## 4. Differential Diagnosis

The clinical features of VKC overlap with a similar condition known as atopic keratoconjunctivitis (AKC), where both represent a severe ocular allergic reaction. AKC affects all ages but has a peak incidence in the fourth and fifth decades of life and is characterized by chronic inflammation that involves the conjunctiva and cornea, where patients typically have concurrent systemic atopic diseases [71]; in fact, more than 95% of AKC patients have eczema, and 87% have a history of asthma [72]. AKC differs from VKC in that there is significant eyelid/dermal involvement that presents as thickened and fissured eyelids with an eczematous, erythematous appearance [71,73]. Corneal complications are common in AKC, ranging from punctate keratitis to the development of persistent epithelial defects and plaque formation [73].

## 5. Ocular Involvement: Signs and Symptoms

Patients with VKC present with complaints of intense ocular pruritis, which could be attributed to the interaction between histamine and histamine receptor 1 expressed on the conjunctival sensory nerve fibers [74,75]. The interaction between IL-31, a cytokine released by traumatized ocular surface epithelial cells, degranulated mast cells, eosinophils, and Th2 cells and their receptors expressed on sensory nerve fibers in the conjunctiva are also responsible for this intense itch sensation [76,77]. Additionally, the chronic itch sensation experienced by individuals with VKC could also be attributed to IL-4 and IL-13 [78]. Frequent eye rubbing secondary to chronic itching is a risk factor for the development of corneal ectasia, which is a well-reported complication of chronic VKC [9,77,79]. The incidence of ectasia development has been reported to be as high as 26.8%, with higher rates of incidence being associated with chronic disease and those of male gender [80]. Patients with the limbal form of the disease are reported to have an increased incidence of keratoconus compared to the other forms of the disease [81]. The bulbar conjunctival hyperemia and perilimbal chemosis observed during clinical evaluation of the ocular surface are clinical signs attributed to the action of mediators derived from mast cells and Th2 cells. Mast cell-derived histamine interacts with histamine receptor 1 (H1R) expressed on blood vessels in the conjunctiva to induce vasodilation and capillary leakage, which manifests as conjunctival hyperemia and chemosis, respectively [82,83]. Similar to the effect of histamine on conjunctival blood vessels, leukotrienes mediate the dilation and permeability of conjunctival blood vessels, which presents as conjunctival hyperemia and chemosis in individuals with VKC, respectively [4]. Furthermore, the conjunctival chemosis and hyperemia in individuals with VKC could be attributed to the action of PGE2 on vascular smooth muscle cells, which leads to increased vascular leakage and vasodilation [29,84,85]. PGD2 induces the dilation and increased permeability of blood vessels in the conjunctiva [84,86] and intensifies the ocular itch sensation [29,87]. Additionally, the binding of IL-4 to its receptors expressed on conjunctival vascular endothelial cells can induce the upregulation of vascular cell adhesion molecule 1 (VCAM-1), which culminates in vasodilation and vasopermeability, observed as conjunctival hyperemia and conjunctiva chemosis, respectively [88,89]. The complaint of mucus discharge could be attributed to the action of IL-13 and histamine. IL-13 is a pleiotropic cytokine secreted by Th2 cells and degranulated mast cells. It can induce the hyperplasia of goblet cells located in the conjunctival epithelium, as well as induce the hypersecretion of mucin from these activated conjunctival goblet cells [4,74,90]. Furthermore, IL-9 also induces the hyperplasia of goblet cells [91]. Additionally, mucoid discharge has been shown to occur when histamine binds to histamine receptors expressed on goblet cells [74,92]. Thus, Th2- and Th9-derived cytokines are contributors to the complaint of mucoid discharge.

**Figure 1:**
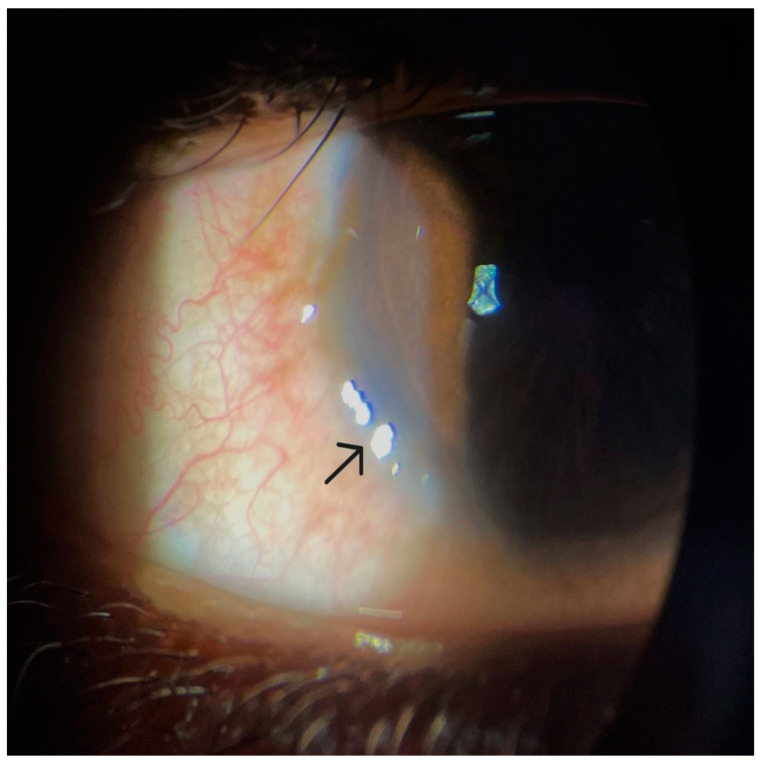
Slit-lamp examination of a right eye that reveals limbal VKC with the presence of perilimbal chemosis and gelatinous limbal papillae (black arrow). Note the hyper-reflectivity in the area of the gelatinous limbal papillae. This patient had previously been treated with 1.0% prednisolone acetate.

**Figure 2:**
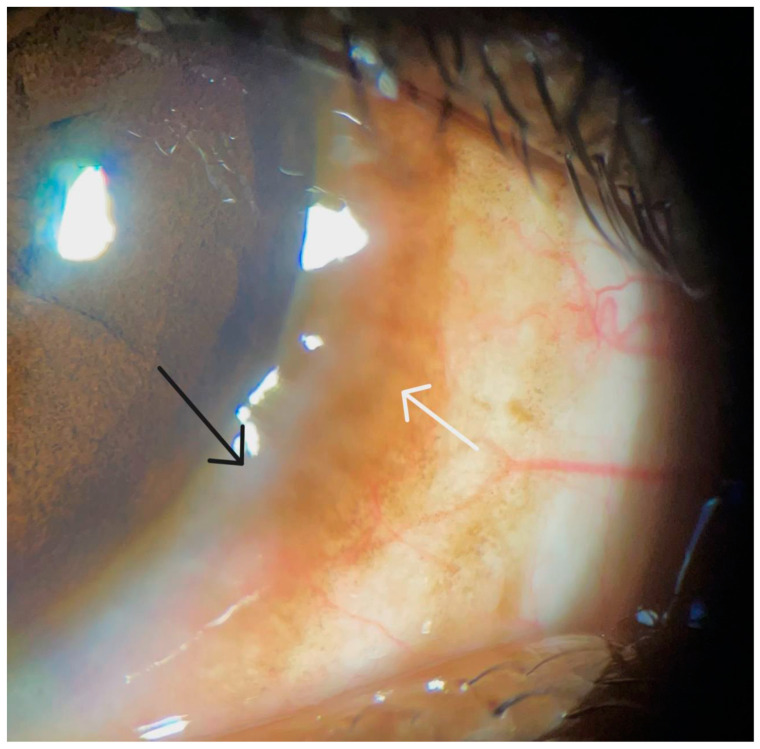
Slit-lamp examination of a left eye that reveals corneal involvement in the form of pseudogerontoxon (black arrow) and perilimbal bulbar conjunctival hyperpigmentation (white arrow).

Ocular involvement in VKC primarily includes changes in the conjunctiva and limbus. The hallmark sign of VKC is papillary hyperplasia of varying degrees and severity, which can affect the upper tarsal conjunctiva and the limbal bulbar conjunctiva [8,93]. Conjunctival fibroblasts express receptors for histamine, IL-4, and IL-13. These mediators act on their cognate receptors expressed on conjunctival fibroblasts to induce hyperproliferation of conjunctival fibroblasts, as well as to promote excess production of extracellular matrix proteins from these fibroblasts [3,4,94]. Additionally, IL-9 secreted by Th2 cells, Th9 cells, eosinophils, and mast cells can synergize with IL-4 in promoting the remodeling of the conjunctival tissue [95,96]. Therefore, activated conjunctival fibroblasts in VKC patients promote fibroproliferative changes and tissue remodeling in the conjunctiva, which is observed as papillae on the palpebral conjunctiva [4,8,93]. Histological examination of papillae in VKC reveals hyperplastic epithelium with a fibrovascular core and the presence of a massive influx of inflammatory cells in the subepithelial layer of the conjunctival tissue [5,8,93]. These inflammatory cells present in a characteristic distribution where lymphocytes localize to the center of the papillae and eosinophils localize to the periphery of the papillae [93]. This massive cell infiltration and hypervascularization, along with hyperproliferation of the conjunctival fibroblasts, results in the appearance of the giant papillae that are seen in VKC. The papillae can vary in size from 0.1 to 5.0 mm, where those larger than 1.0 mm are classified as giant papillae, and, when confluent, give the classic “cobblestone” appearance [5]. This papillary hypertrophy typically manifests in the superior palpebral conjunctiva in VKC, and inferior palpebral conjunctiva in AKC [71,73]. Limbal VKC is characterized by the presence of confluent, gelatinous papillae located mainly on the limbal region of the bulbar conjunctiva (Figure 1). Increased pigmentation of the perilimbal intrapalpebral conjunctiva can occur in non-Caucasian patients with VKC (Figure 2) [5,97,98,99]. The color of the pigment can vary from light golden-brown to dark brownish-black and can be seen in active or quiescent disease forms [98,99]. There is an abundance of melanocytes and mast cells within the limbal region of the bulbar conjunctiva [99]. It is postulated that immune molecules such as histamine and stem cell factors can activate histamine receptor 2 and CD117 expressed on melanocytes located within the limbal region of the bulbar conjunctiva, respectively. This interaction induces the melanocytes to produce excess melanin, which causes pigmentary changes within the intrapalpebral bulbar conjunctiva [99]. The presence of perilimbal hyperpigmentation can help confirm the diagnosis of VKC, especially when signs and symptoms are subtle, since it appears to be a consistent finding in certain populations with VKC [99,100].

While the conjunctiva is the primary site of ocular involvement in VKC, the corneal tissue may become compromised in response to the chronic and exaggerated immune response, leading to permanent vision loss in some cases. Corneal involvement occurs in around 50% of patients and is common in the tarsal, mixed, and perennial forms of the disease [9,101]. Corneal changes include punctate epithelial keratitis, macroerosions, neovascularization, microbial keratitis, shield ulcers, scarring, and plaque formation [1,101,102]. IL-5 derived from degranulating mast cells and Th2 cells recruits and activates eosinophil, a granulocyte that participates in the inflammation-associated damage and remodeling of the ocular surface observed in chronic forms of ocular allergy such as vernal keratoconjunctivitis [103,104,105]. The pathogenesis of corneal involvement is secondary to the toxic effects from mediators released during the degranulation of eosinophils in the conjunctiva, including the eosinophilic cationic protein (ECP), major basic protein (MBP), and matrix metalloproteinase-9 (MMP-9) [3,9]. These mediators induce degradation of the extracellular matrix proteins present in the corneal epithelial basement membrane. This results in the persistent epithelial keratopathy that is seen in VKC [102,106]. Additionally, IL-9 can interact with epithelial cells of the ocular surface to cause a breach of the barrier function of the epithelium, which culminates in the development of punctate epithelial erosions due to the compromised ocular surface epithelium [31]. Persistent corneal epithelial compromise extending into the Bowman’s layer, along with degradation of the collagen type 1 present in the corneal stroma, can lead to an oval-shaped defect with elevated margins and can present as a shield ulcer, a vision-threatening complication of VKC [9,107]. Patients may also present with pseudogerontoxon, which appears as a gelatinous grey-white segment in the peripheral corneal stroma and is the result of lipid deposition from chronic increased limbal vasopermeability (Figure 2) [1,101]. It is of note that vascular endothelial cells express receptors for IL-4 and histamine. As such, the persistent IL-4 activation of these endothelial cells on limbal blood vessels results in the upregulation of VCAM-1 on these vascular endothelial cells, which culminates in a breach of the endothelial barrier function [88,89].

## 6. Management of VKC

The main goals of VKC treatment include controlling acute symptoms and preventing recurrent disease. Currently, there are no well-established treatment guidelines for VKC, and options are individualized based on disease stage, severity, and duration [108]. Because the immunopathogenesis of VKC is multifactorial, the use of a combination of agents with more direct effects on the inflammatory processes is ideal to control acute symptoms and prevent recurrent disease [4]. Non-pharmacological disease management includes the identification and avoidance of specific allergens, and pharmacological therapy includes antihistamines, mast cell stabilizers, and non-steroidal anti-inflammatory drugs (NSAIDs) [4,108,109]. Mild disease is typically managed with topical mast cell stabilizers, antihistamines, or dual-acting agents [5,110,111]. Mast cell stabilizers work by inhibiting mast cell degranulation, which thereby prohibits the release of pro-inflammatory mediators [4,6]. Mast cell stabilizers have also been shown to act on additional cells involved in ocular allergy, including neutrophils, macrophages, eosinophils, and monocytes [112]. Antihistamines block histamine from interacting with their receptors on immune and non-immune cells [4,6]. Most antihistamines target the H1 receptor to provide acute symptomatic relief, whereas mast cell stabilizers exhibit a slow onset of action and aid in chronic management [110]. Dual-acting agents, which contain both mast cell stabilizers and antihistamines, offer the advantage of providing rapid symptomatic relief, coupled with long-term mast cell stabilization [4,6,110]. These agents are well tolerated, and dual-therapy agents are generally preferred over monotherapy with mast cell stabilizers or antihistamines alone [112]. Topical non-steroidal anti-inflammatory drugs (NSAIDs) work by inhibiting cyclooxygenase (COX)-1 and COX-2 enzymes, which thereby prohibits the release of prostaglandin E2 and I2 [4,110]. Prostaglandins are reported to have pruritogenic properties; therefore, the inhibition of these prostaglandins allows for the mitigation of ocular itching [1,113]. While NSAIDs provide symptomatic relief, they do not have any effect on the papillary hyperplasia or limbal changes seen in VKC [113,114]. Additionally, due to the side effects of corneal toxicity from these agents, prolonged use is ill-advised, especially if patients present with corneal involvement. While these agents provide symptomatic relief, they do not fully target the complex immune processes involved in VKC. 

Moderate to severe forms of the disease require effective therapy to target the specific immune responses involved in VKC. Anti-inflammatory agents that are more effective include corticosteroids; however, these agents are associated with potentially serious adverse effects, including cataract formation, increased intraocular pressure, delayed wound healing, and increased susceptibility to infection [29]. In severe VKC, topical steroids are administered for a greater duration due to the intensity of the inflammation in these patients [22]. While long-term and indiscriminate use of topical corticosteroids is ill-advised, a “pulse” regimen of four drops per day for 5 days is preferred to manage acute episodes of VKC, and limiting longer treatment regimens to between one and three weeks with a slow taper is preferred for chronic episodes of VKC [109]. Low-potency steroids are initially used and include agents such as loteprednol and fluorometholone. In cases where low-potency steroids fail, higher-potency steroids can be administered, including agents such as prednisolone, dexamethasone, and diflurprednate. While corticosteroids are efficacious in controlling signs and symptoms of VKC, steroid-resistant forms of VKC exist and may necessitate alternative therapeutic management [115,116]. Additionally, VKC is associated with frequent recurrences of inflammation and often requires steroid-sparing agents to manage the condition [115,116].

Topical immunomodulators play a crucial role in the management of VKC as they are effective and have a better safety profile in the treatment of severe and recalcitrant disease [4]. These immunomodulators, such as calcineurin inhibitors, work by modulating the immune response and have achieved breakthrough results in the treatment of severe disease. Immunomodulators exert their therapeutic effects by binding to immunophilins (cyclophilin and FK506-binding protein) to form an immunomodulator–immunophilin complex that binds to calcineurin [117,118]. The binding of this complex to calcineurin interferes with the signaling pathway that is responsible for the proliferation of T cells. Cyclosporin binds to cyclophilin to form the cyclosporin–cyclophilin complex, which binds to calcineurin, thereby preventing calcineurin from the dephosphorylating nuclear factor of activated T cells (NFAT). This action blocks the migration of the cytoplasmic NFAT into the nucleus [118,119,120]. It is important to note that calcineurin is required for the activation of NFAT, and this is followed by the migration of the activated NFAT into the nucleus, where it binds to the regulatory region of T cell-derived cytokines to mediate transcription of genes that encode for IL-2 and other T cell-derived cytokines [118,119]. While corticosteroids are often used for short-term relief of acute episodes, calcineurin inhibitors are employed for long-term immunosuppression of chronic episodes. Specifically, calcineurin inhibitors, such as cyclosporine and tacrolimus, modulate the immune response by inhibiting the IL-2-mediated clonal expansion of Th2 cells, thereby inhibiting the release of Th2 cell-derived cytokines [4,109] such as IL-3 and IL-9, which have been shown to promote the growth and development of mast cells in ocular allergies [121]. Topical cyclosporine A (CsA) is effective in controlling inflammation of the ocular surface as it has been found to reduce the number of neutrophils, eosinophils, and lymphocytes infiltrating the conjunctiva, and is efficacious even at low concentrations such as 0.05% [4,111,122]. CsA has been shown to inhibit mast cell-mediated cytokine production by inhibiting Th2 lymphocyte proliferation, IL-2 production, and TNF-alpha production, thereby leading to an inhibitory effect on the development of ocular allergies [122]. Additional effects of CsA include inhibiting IL-5 production, which thereby prevents the recruitment of eosinophils, and ultimately inhibits the release of eosinophil-derived mediators such as eosinophil major basic protein to the site of ocular allergies [1,4,111]. CsA has been shown to significantly reduce tear levels of IL-4, IL-5, IL-17, TNF-alpha, IFN-gamma, and eotaxin, which have been shown to be increased in the tear films of patients with VKC [122,123]. Different concentrations of CsA range from 0.05% to 2.0% and are available in select countries [112]. Commercially available forms include 0.05%, which is authorized for dry eye disease, and 0.1%, which is authorized for severe VKC. Both the low- and high-dose ends of the spectrum, 0.1% and 2.0%, respectively, have been shown to have comparable efficacy and safety profiles in treating severe VKC, where both dosages were found to enable a substantial reduction in the use of topical corticosteroids [124]. In a study evaluating the efficacy of 0.1% CsA in a cationic emulsion versus a hospital preparation of 2.0% CsA via dilution of an intravenous preparation, both dosages were found to induce comparable improvements in clinical signs and patient symptoms [124]. Interestingly, the 0.1% CsA group was able to control their symptoms with a lower number of daily instillations (two drops per day on inclusion, then one drop per day) compared to the 2.0% CsA group (three drops per day on inclusion, then two drops per day). This is likely due to the fact that CsA is a lipophilic substance, making it insoluble in water [125]. Because the 0.1% CsA formulation was delivered in a lipid-based system, there was increased retention on the ocular surface, allowing for a lower dose to provide similar therapeutic effects [124]. In a study [123] evaluating the efficacy of 0.05% CsA in VKC, patients were treated with a loading dose of four times a day for two weeks, followed by gradual tapering to three times a day for a week, then twice a day for the next week, once a day for a week, and finally a maintenance dose of one drop per day until the final follow up. The results of this study revealed a beneficial effect of 0.05% CsA in reducing signs and symptoms as early as two weeks, which was maintained after three months [123]. CsA has been shown to be efficacious in treating signs and symptoms of VKC: specifically, 91% of patients treated with 2% CsA four times daily showed decreased symptoms, and 66% showed improvement in clinical signs of VKC [126]. It has also been observed through the VEKTIS trial that one month of treatment with 0.1% CsA four times daily improved symptoms and quality of life, and was well tolerated in children and adults with severe VKC [127,128]. This finding was additionally supported by the NOVATIVE trial, which evaluated the efficacy of both 0.05% and 0.1% CsA [129]. This trial found that improvement was most notable with the 0.1% CsA, where patients had improved signs and symptoms as early as 1 week, which continued throughout the first month of treatment [130]. A pooled analysis of the data from both studies demonstrated that both high and low dosages of CsA were safely tolerated, where the most frequent adverse events were mild to moderate pain at the instillation site (high dose, 9.4%; low dose, 7.5%), and pruritus (high dose, 5.2%; low dose, 7.5%) [129]. Recommendations from the Expert Working Group in Asia (MOVIA) suggest considering the initiation of topical 0.1% CsA for patients with moderate to severe or persistent VKC [112]. This finding is also supported by the European Expert Consensus (Eur-VKC Group), who recommend topical CsA in order to provide a corticosteroid-sparing effect [114]. The MOVIA group recommend initiating CsA as a first line of treatment for patients presenting with moderate to severe VKC, and only prescribing corticosteroids as a short-pulse treatment if there is an inadequate response to the CsA, or if there are corneal complications. In contrast, the EUR-VKC Group recommend starting with short-pulse corticosteroids before CsA in the treatment sequence. While there is no consensus on the specific concentration or dosage of CsA, both groups suggest the use of CsA for long-term control [112,114]. This long-term control was demonstrated in a case report of a 12-year-old male with a recurrent vernal shield ulcer after discontinuing CsA [131]. Once 0.1% CsA was reinitiated, there was complete resolution of his shield ulcer with no reported recurrence. This benefit was additionally observed in a case report of multiple patients, where initiation of topical CsA was found to cause marked improvements in corneal shield ulcers [132]. All patients in this case were treated with 0.1% CsA, dosed eight times per day, and were concurrently treated with a corticosteroid. Gradual tapering of the treatment was initiated once improvement was noted, and topical CsA was maintained at twice or four times daily to prevent recurrent disease. CsA has been shown to be safe and effective and is used in varying concentrations, on-label and off-label, for the treatment of VKC [109,127,129].

Tacrolimus is another calcineurin inhibitor that works to modulate the inflammatory response by binding to FK506-binding protein (FKBP) to form a tacrolimus–FKBP complex that binds to calcineurin. This interferes with the ability of calcineurin to dephosphorylate NFAT [120]. Thus, tacrolimus suppresses the production of cytokines such as IL-2, IL-4, and IL-5 [133] and also suppresses histamine release by mast cells [134]. This agent has a long history of being used as an immunosuppressant after organ transplantation [109] and is significantly more potent than cyclosporine [134,135]. This increased potency of tacrolimus is beneficial in treating cases refractory to cyclosporine [136]. Tacrolimus drops and ointments come in varying concentrations, ranging from 0.005% to 0.1% [13,109,137], with 0.1% being the most common [133]. Tacrolimus 0.1% eye drops are not FDA-approved and are currently used off-label for the treatment of VKC. In a study evaluating the safety and efficacy of 0.1% tacrolimus in pediatric patients who responded poorly to CsA, significant improvements were noted in clinical signs including marked improvements in giant papillae in all forms of the disease [138]. This finding was additionally supported by a study evaluating the effect of 0.03% tacrolimus in patients who were unsuccessfully treated with CsA and corticosteroids [139]. In this study, the patients experienced significant reductions in their signs and symptoms after 4 weeks of treatment with the following dosing schedule: topical tacrolimus ointment 0.03% twice daily for 8 weeks, and then once daily for 2 months, followed by three times daily for 8 weeks. Resolution of giant papillae and corneal lesions was noted within 8 weeks of treatment. In a study evaluating the efficacy of 0.03% versus 0.1% tacrolimus in recalcitrant VKC, both strengths were effective in treating VKC, where 88% of patients receiving 0.03% and 94% of patients receiving 0.1% experienced significant improvements in their clinical signs and symptoms [137]. Resolution of the papillary component of VKC depends on the strength of treatment, where a higher strength (0.1%) provides increased efficacy in controlling papillary hyperplasia [137]. This effect was also observed in another study evaluating the efficacy of 0.03% tacrolimus in VKC [134], which reported a significant reduction in papillae in the tacrolimus-treated group compared to the control group (fluorometholone 0.1%). In another study evaluating the efficacy of 0.1% tacrolimus, patients previously treated with 2 months of 0.1% cyclosporine only experienced significant resolution of their papillae with administration of 0.1% tacrolimus for 1 month [140]. Patients treated with tacrolimus can exhibit improvements in their clinical signs and symptoms as early as three days [141], where the most common adverse events reported are transient burning [137,140,142,143] and ocular stinging [134,137]. Both cyclosporin and tacrolimus reduce the transcription of cytokine genes required for encoding T cell-derived cytokines [119,120] and are effective immunomodulators for controlling the inflammatory reaction seen in VKC. In a study evaluating the efficacy of 0.03% tacrolimus versus 2% CsA, tacrolimus was reported to be more effective in treating signs and symptoms of VKC compared to 2% CsA [144]. Interestingly, another study comparing 0.1% tacrolimus with 2% CsA revealed that both were equally effective in the treatment of VKC [145]. Currently, there are no established protocols for VKC treatment using immunomodulators. A treatment protocol that has been proposed includes initiating 0.03% tacrolimus as a first line of therapy for severe VKC, increasing the dose to 0.1% if no improvement is noted, or for cases that do not show improvement with corticosteroid therapy [146]. Patients who received tacrolimus as first-line treatment were shown to have shorter treatment periods and follow-up durations compared to patients who received tacrolimus as second-line treatment. Monitoring these patients closely every 2–4 weeks initially is advised, as tacrolimus can take up to one month to take effect. Gradual tapering of the treatment is initiated once improvement in clinical signs and symptoms is noted [146]. In a study evaluating the efficacy and safety of prolonged treatment of severe VKC with topical 0.1% tacrolimus, it was demonstrated that 2 years of treatment was effective in improving clinical scores and remission rates, with limited adverse effects compared to a group treated for 6 months [133]. In fact, no adverse events occurred from the 12th to the 24th month of treatment use. Another study evaluating the safety of 0.1% tacrolimus in the treatment of refractory AKC for 48 months demonstrated similar findings, with complete clinical resolution and remission of disease in the absence of other medications, and with mild adverse effects that were limited to a mild burning sensation [147]. These findings suggest that patients with severe forms of VKC likely require therapy for longer periods of time in order to prevent recurrences, and they highlight the safety of long-term application.

**Figure 3:**
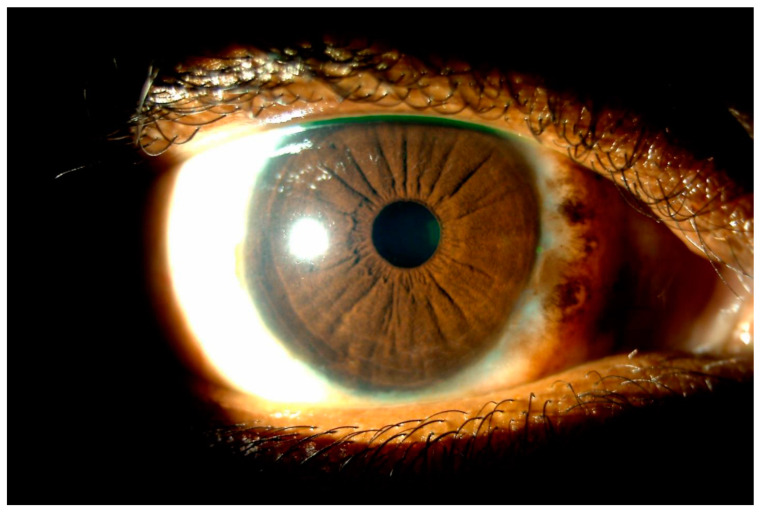
Slit-lamp examination of a right eye that reveals resolution of the limbal papillae with no active signs of vernal keratoconjunctivitis after initiation of 0.1% CsA for 6 weeks.

Both CsA and tacrolimus have been used off-label for the treatment of severe or steroid-intolerant disease. The use of a combination of immunomodulators was first evaluated in the treatment of patients with steroid-dependent AKC [148]. The results from this study demonstrated a reduction in the number of recurrences, while limiting the use of corticosteroids. In a study evaluating the role of combined immunomodulator therapy in severe steroid-intolerant VKC, the use of CsA and tacrolimus had a synergistic effect and provided rapid resolution of signs and symptoms once the tacrolimus was introduced into the treatment regimen [115]. The patients in this study were initially treated with 0.1% CsA four times daily, which showed significant improvement within 2 weeks of therapy; however, the response was considered suboptimal, which prompted the inclusion of a 0.03% tacrolimus ointment twice daily to the treatment regimen. The addition of tacrolimus resulted in significant improvement and resolution of clinical signs and symptoms including itching, photophobia, discharge, tarsal papillae, and punctate keratopathy. The improved response is likely due to the higher potency of tacrolimus. It is also worth noting that while both drugs inhibit the calcineurin receptor, they do so by binding to different sites (CsA binds to cyclophilin A, and tacrolimus binds to FKBP12). Combined immunomodulator therapy allows for the rapid control of symptoms and should be considered in cases of severe inflammation [115,148]. While there have been no head-to-head randomized controlled studies of 0.1% tacrolimus and cyclosporine, varying concentrations of both agents, used both on-label and off-label, are safe and effective in the treatment of VKC. However, although these topical immunomodulators are effective in treating and controlling VKC, it is suggested to reserve cyclosporine and tacrolimus for severe or refractory forms of VKC, or for cases where corticosteroids are contraindicated [111,137,149]. Patients that do not respond to corticosteroids (Figure 1) can access therapeutic benefits with the use of immunomodulators (Figure 3).

## 7. Future Directions

In recent years, therapy employing biologics and monoclonal antibodies has been developed to treat allergic diseases [150]. Such therapies are currently not approved for allergic eye disease; however, it has been reported that the off-label use of these agents provides promising results in the treatment of ocular allergic disease. Specifically, omalizumab, a humanized monoclonal anti-IgE antibody, appears to offer therapeutic relief in patients with severe VKC by targeting the IgE-mediated pathophysiology of the condition [108,151]. Larger controlled clinical trials are needed to establish the role of biologics and monoclonal antibodies, and to determine the appropriate dosage and administration of these agents in the treatment of VKC. Additional efforts in research are needed to identify the specific pathogenesis of VKC to allow the formulation of new molecules that target the diverse pathogenic mechanisms of this condition. Additionally, practitioners would benefit from established diagnostic criteria and treatment guidelines, especially for moderate, severe, and refractory cases of VKC.

## 8. Conclusions

VKC is a sight-threatening chronic inflammatory condition that primarily affects pre-pubescent males in warm climates. The conjunctiva and cornea are the major ocular sites affected, where prolonged and chronic inflammation can result in impaired visual function and quality of life. This review highlighted the importance of understanding the fibroproliferative changes that occur in VKC and selecting appropriate therapy to control the inflammatory cascade. The VKC treatment armamentarium includes both on- and off-label therapeutic options, including the use of immunomodulators in severe and refractory cases of VKC. Correct management is imperative to prevent adverse effects and ocular complications associated with this challenging condition of the ocular surface.

## Data Availability

No new data were created or analyzed in this study. Data sharing is not applicable to this article.
